# Sarcoplasmic Reticulum from Horse Gluteal Muscle Is Poised for Enhanced Calcium Transport

**DOI:** 10.3390/vetsci8120289

**Published:** 2021-11-23

**Authors:** Joseph M. Autry, Bengt Svensson, Samuel F. Carlson, Zhenhui Chen, Razvan L. Cornea, David D. Thomas, Stephanie J. Valberg

**Affiliations:** 1 Department of Biochemistry, Molecular Biology, and Biophysics, University of Minnesota, Minneapolis, MN 55455, USA; bsven@ddt.umn.edu (B.S.); sacarlson@mcw.edu (S.F.C.); corne002@umn.edu (R.L.C.); ddt@umn.edu (D.D.T.); 2 Krannert Institute of Cardiology, Department of Medicine, Indiana University School of Medicine, Indianapolis, IN 46202, USA; zhechen@iu.edu; 3 McPhail Equine Performance Center, Department of Large Animal Clinical Sciences, College of Veterinary Medicine, Michigan State University, East Lansing, MI 48823, USA

**Keywords:** calcium regulation, calsequestrin, comparative biochemistry, excitation–contraction coupling, exertional rhabdomyolysis, intraluminal protein, ion-motive ATPase, membrane vesicles, regulatory subunit, skeletal muscle

## Abstract

We have analyzed the enzymatic activity of the sarcoplasmic reticulum (SR) Ca^2+^-transporting ATPase (SERCA) from the horse gluteal muscle. Horses are bred for peak athletic performance yet exhibit a high incidence of exertional rhabdomyolysis, with elevated levels of cytosolic Ca^2+^ proposed as a correlative linkage. We recently reported an improved protocol for isolating SR vesicles from horse muscle; these horse SR vesicles contain an abundant level of SERCA and only trace-levels of sarcolipin (SLN), the inhibitory peptide subunit of SERCA in mammalian fast-twitch skeletal muscle. Here, we report that the in vitro Ca^2+^ transport rate of horse SR vesicles is 2.3 ± 0.7-fold greater than rabbit SR vesicles, which express close to equimolar levels of SERCA and SLN. This suggests that horse myofibers exhibit an enhanced SR Ca^2+^ transport rate and increased luminal Ca^2+^ stores in vivo. Using the densitometry of Coomassie-stained SDS-PAGE gels, we determined that horse SR vesicles express an abundant level of the luminal SR Ca^2+^ storage protein calsequestrin (CASQ), with a CASQ-to-SERCA ratio about double that in rabbit SR vesicles. Thus, we propose that SR Ca^2+^ cycling in horse myofibers is enhanced by a reduced SLN inhibition of SERCA and by an abundant expression of CASQ. Together, these results suggest that horse muscle contractility and susceptibility to exertional rhabdomyolysis are promoted by enhanced SR Ca^2+^ uptake and luminal Ca^2+^ storage.

## 1. Introduction

Horses are highly susceptible to muscle exertional rhabdomyolysis from a variety of causes, including glycogen storage disorders, malignant hyperthermia, and abnormalities in cytosolic Ca^2+^ regulation [1]. The horse species has been bred selectively for thousands of years to achieve a remarkable athletic ability, in part conferred by a naturally high proportion (75–95%) of fast-twitch myofibers in the locomotor muscles that provide powerful contraction and rapid running [2]. Recurrent exertional rhabdomyolysis (RER) is one of the most common causes of poor performance and economic loss in Thoroughbred racehorses [3,4]. The molecular etiology of RER in Thoroughbred racehorses has been proposed to involve defects in excitation–contraction coupling, SR Ca^2+^ cycling, electron transport, and mitochondrial protein translation [1,5,6,7,8].

Valberg et al. [9] used whole-transcriptome RNA shotgun sequencing (RNA-seq) to determine the amino acid sequence and transcription level of Ca^2+^ regulatory proteins in Thoroughbred muscle. These proteins include the sarcoplasmic reticulum (SR) Ca^2+^-transporting ATPase protein (SERCA = 110 kDa), the regulatory peptide subunits sarcolipin (SLN = 3.3 kDa), and phospholamban (PLN = 5.2 kDa), plus the luminal Ca^2+^-storage protein calsequestrin (CASQ ~ 55 kDa). RNA-seq determined that (i) the *SLN* transcript is the predominant regulatory peptide expressed in horse muscle, as compared to the *PLN* transcript, and (ii) the *SLN* transcript is expressed at a many-fold greater level than the *ATP2A1* transcript, which produces the SERCA1 protein expressed in fast-twitch skeletal muscle (hereafter, SERCA1 will be referred to as ‘SERCA’) [9,10]. RNA-seq also determined that the gene expression of the *CASQ1* transcript, which produces the fast- and slow-twitch skeletal muscle protein isoform (hereafter referred to as CASQ) is downregulated in the gluteal muscle of male or female horses with RER, as compared to healthy male or female horses (controls). Furthermore, expression of the *CASQ* transcript was downregulated to a greater extent in the gluteal muscle of male horses with RER compared to female horses with RER [9]. Thus, RNA-seq results identified potential molecular mechanisms that contribute to the high performance of horse muscle and suggested a possible need for gender-specific pharmacophore therapies for horse RER susceptibility.

We recently developed an improved protocol for the purification of SR vesicles from horse muscle, which provides 5–25-fold greater Ca^2+^-activated ATPase activity than previously reported for SERCA in horse muscle SR vesicles [11]. This new horse SR prep allowed for an improved characterization of the protein profile of horse SR using quantitative immunoblotting and Stains-all in-gel staining [11]. The RNA-seq data demonstrated the supra-abundant expression of the *SLN* transcript in horse muscle, yet quantitative immunoblotting detected only a trace level of SLN peptide in horse SR vesicles, as compared to SERCA protein (0.06 SLN/SERCA mol/mol) [9,10,11]. SLN is the primary regulatory peptide of SERCA expressed in skeletal muscles from larger mammals, e.g., rabbits, dogs, pigs, and humans [12,13,14,15]. SLN protein expression has been reported at a significant level in only a few mouse skeletal muscles, for example, in the slow-twitch soleus muscle of adult mice, in the fast-twitch gastrocnemius muscle of aging mice, and in some other muscle types in atrophy mouse models [16]. In mice, other regulatory peptides have been shown to regulate SERCA activity. Therefore, the use of SLN knockout transgenic mice as experimental model muscles to study the lack of SLN is limited. Thus, an investigation of Ca^2+^ uptake activity using a SR skeletal muscle system that lacks known SERCA inhibitors, i.e., the minimal or insignificant expression of SLN, PLB, and MRLN, has been limited to in vitro assays of Ca^2+^-activated ATPase activity by SR vesicles purified from horse gluteus [10,11].

The general consensus is that SLN inhibits SERCA activity via multiple enzymatic mechanisms: by decreasing the maximal velocity (V_max_), by decreasing the apparent Ca^2+^ binding affinity (1/K_Ca_), by decreasing ATP binding affinity (1/K_ATP_), and by decreasing the number of Ca^2+^ ions transported per ATP molecule hydrolyzed (coupling ratio) below the optimal Ca^2+^/ATP coupling ratio of two [17,18,19,20,21,22,23]. The SLN inhibition of SERCA activity is relieved in part by SLN phosphorylation or de-acylation [24,25,26]. In a mouse slow-twitch muscle (soleus and red gastrocnemius), the genetic knockout of SLN results in an enhanced ATP-dependent Ca^2+^ uptake by SERCA [27]. In patients with atrial fibrillation (AFib) or heart failure with preserved ejection fraction (HFpEF), a decreased expression of SLN correlates with an increased Ca^2+^ uptake in atrial SR; however, it is unknown if decreased SLN inhibition and concomitant SERCA activation are compensatory or causative in the progression of human cardiovascular disease [28,29].

Aberrant Ca^2+^ signaling from SR Ca^2+^ stores in skeletal myocytes has been identified in pig malignant hyperthermia (MH) due to a natural mutation of the ryanodine receptor SR Ca^2+^ release channel (RYR) and also in a mouse genetic model of increased sarcolemmal Ca^2+^ entry that leads to an SR store overload-induced Ca^2+^ release (SOICR) through RYR [30,31,32]. Mutations in the luminal Ca^2+^ storage protein calsequestrin (CASQ) result in catecholaminergic polymorphic ventricular tachycardia (CPVT) [33,34]. Thus, the rate of SERCA Ca^2+^ transport activity, the level of SLN inhibition, and the amount of luminal SR Ca^2+^ are critical for the coordinated tuning of Ca^2+^ cycling kinetics in myocytes, thereby matching the physiological requirements for the contraction and relaxation of each specific muscle type.

In the present study, we used biochemical approaches to investigate Ca^2+^ transport regulation in horse gluteus, a predominant locomotor muscle. The enzymatic properties of SERCA in horse SR vesicles were compared to SERCA in rabbit SR vesicles, which are the standard experimental model for examining SR function, as previously discussed [11]. These studies identified a high rate of SERCA Ca^2+^ transport activity (possibly due to the decreased SLN expression) and a higher total luminal Ca^2+^ accumulation (possibly due to the enhanced CASQ expression) in horse SR. These findings were interpreted in light of the high susceptibility of horses to exertional rhabdomyolysis. We propose that comparative studies of biochemical regulation of SR enzymes will increase the broader understanding of the selective adaptation of horse muscle, with a specific focus on species-dependent performance and disease.

## 2. Materials and Methods

### 2.1. Materials

The Enzyme Commission (EC) number of SERCA is 7.2.2.10 [35]. Proteinase K (EC 3.4.21.64) and standard chemicals were purchased from Sigma-Aldrich Corporation (St. Louis, MO, USA). The ^45^Ca (CaCl_2_) isotope was purchased from New England Nuclear Corporation (Billerica, MA, USA).

### 2.2. Animals

Muscle samples were obtained from four horses (*Equus caballus*) that were donated to the University of Minnesota for euthanasia due to orthopedic disease; these horses had healthy musculoskeletal systems. Table S1 lists details on the following horses examined in this study: three castrated males (two Quarter Horses and one Thoroughbred) and one female (Quarter Horse). The horses examined in this study were pastured horses donated to the University because of chronic lameness. They had no history of muscle disease and were of ages ranging from 10 to 18 years (Table S1). The horse owners provided written consent for obtaining muscle samples for this research.

Muscle samples were also obtained from six New Zealand White rabbits that were provided by the University of Minnesota Research Animal Resources Facility. The New Zealand White rabbits examined in this study were junior does of age ≤6 mo.

The University of Minnesota Research Animal Resources Facility complies with the USDA Animal Welfare Act Regulations and the NIH Public Health Service Policy on Humane Care and Use (Animal Welfare Assurance approval A3456-01 via the NIH Office of Laboratory Animal Welfare). The University of Minnesota received accreditation renewal from the Association for Assessment and Accreditation of Laboratory Animal Care (AAALAC) in November 2015. All animal research was reviewed and approved by the Institutional Animal Care and Use Committee (IACUC) at the University of Minnesota, with IACUC protocol # 1511-33199A for horses and IACUC protocol # 1611-34327A for rabbits. Palliative care and euthanasia protocols [9,10,11] were consistent with guidelines from the American Veterinary Medical Association.

### 2.3. Purification of SR Vesicles from Horse and Rabbit Muscle

SR vesicles from horse gluteus (predominantly fast-twitch myofibers) and rabbit skeletal muscle (fast-twitch muscles pooled from back and legs) were isolated using mechanical homogenization and differential centrifugation. Horse SR vesicles are defined as the pellet from the centrifugation of a gluteal muscle homogenate at 10,000× *g* for 20 min at 4 °C, following an initial clarification spin of 3800× *g* for 20 min at 4 °C. Rabbit SR vesicles are defined as the pellet from the centrifugation of a muscle homogenate (pooled back muscle and hind leg muscle) at 23,000× *g* for 60 min, following initial clarification spins of 4000× *g* for 20 min at 4 °C and 11,800× *g* for 20 min at 4 °C [10,11,36]. The pooled muscle tissue from the rabbit was white colored (indicating a fast-twitch muscle).

### 2.4. SDS-PAGE and Coomassie Densitometry

SDS-PAGE of SR vesicles was performed as reported previously [10,11,37]. Proteins were stained in-gel with Coomassie blue R-250. A GelDoc EZ imaging system with the software Image Lab 5.0 (Bio-Rad Laboratories Incorporated; Hercules, CA, USA) was used to scan Coomassie-stained gels and to quantitate the relative absorbance of each Coomassie-stained protein band. Camera exposure time was optimized to prevent pixel saturation of the absorbance intensity for each Coomassie-stained protein band.

### 2.5. Oxalate-Facilitated ^45^Ca^2+^ Transport Assay

Ca^2+^ transport by SR vesicles was measured using a filtration assay with a ^45^Ca radioactive tracer [37,38]. Assays were conducted at 25 °C with 10 μg of SR protein in a 1-milliliter solution containing 100 mM KCl, 3.3 mM MgCl_2_, 3.0 mM Na_2_ATP, 10 mm K_2_-oxalate, 5 mM NaN_3_, and 50 mM MOPS (pH 7.0). The addition of 2 mM EGTA and 1.8 mM CaCl_2_ (containing trace amounts of ^45^Ca) was used to produce an ionized Ca^2+^ concentration ([Ca^2+^]_i_) of 2.4 µM, i.e., a V_max_ assay with a saturating concentration of substrates Ca^2+^, Mg^2+^, and ATP. Transport assays were started by the addition of protein samples to reaction tubes. Ca^2+^ transport was terminated at serial time intervals by vacuum filtering 100 µL of assay solution (i.e., 1 µg of SR protein) through a HA-type glass-fiber filter with a 0.45-micrometer pore size (Millipore Corporation; Burlington, MA), which was washed twice with 5 mL of ice-cold 150 mM NaCl solution. The loading of ^45^Ca inside SR vesicles was determined by liquid scintillation counting. Background ^45^Ca binding (defined as a glass-fiber filter blank loaded with 1 µg of SR protein in the absence of ATP) was subtracted from the experimental values (the glass-fiber filter sample loaded with 1 µg of SR protein in the presence of ATP) to yield the amount of ATP-dependent Ca^2+^ transport by SR vesicles.

### 2.6. Proteinase K Assay of SERCA Conformational States

Controlled proteolysis by Proteinase K (ProtK) was used to assess the ligand-dependent conformational state of the horse and rabbit SERCA [39,40]. The standard assay solution contained 50 mM NaCl, 0.5 mM MgCl_2_, and 20 mM MOPS (pH 7.0), with an addition of either 0.1 mM CaCl_2_ (to stabilize the E1•2Ca^2+^ biochemical state) or 2 mM EGTA and 1 μM TG (to stabilize the Ca^2+^-free E2•TG biochemical state) [41]. SR vesicles at 500 µg/mL were incubated with 12.5 µg/mL ProtK (40/1 wt/wt protein) for 15 min at 23 °C. Proteolysis was stopped by the addition of ice-cold trichloroacetic acid (TCA at 2.5% *wt/vol*), followed by the addition of a Laemmli sample solution (final concentration of 1.1% lithium dodecyl sulfate). SR samples were electrophoresed through a 4–15% Laemmli gel. Non-proteolyzed proteins and proteolytic fragments were stained in-gel with Coomassie blue. The ProtK-mediated cleavage pattern of SERCA (e.g., the 95 and 83 kDa fragments) was imaged using the in-gel absorbance intensity of Coomassie-stained bands detected using a Bio-Rad GelDoc EZ imaging system.

### 2.7. Experimental Design, Statistical Analysis, and Data Presentation

Biochemical assays were performed using independent SR vesicle preparations from N = 2–4 horses and N = 3–6 rabbits. Scientists were not blinded to sample identity during data acquisition or analysis. Data are reported as mean ± standard error (SEM). Data graphs were generated using Origin 2015 software (OriginLab Corporation; Northampton, MA, USA). For statistical difference determination, we used two-way, unpaired Student’s *t*-test. Significance was accepted at *p* < 0.05.

## 3. Results

### 3.1. Horse SR Vesicles Contain an Abundant Amount of SERCA Protein, Although at a Lower Level Than Rabbit SR Vesicles

**Figure 1:**
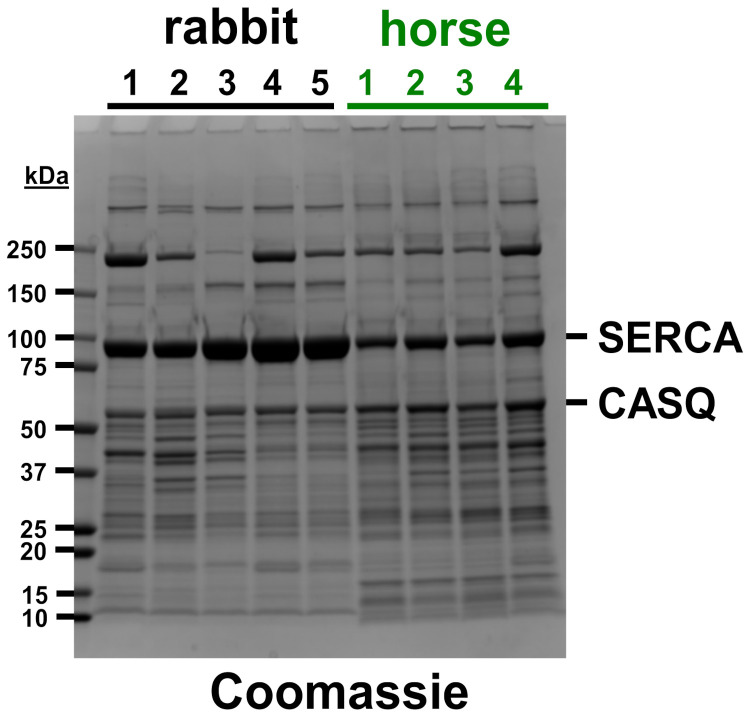
Coomassie gel of rabbit and horse SR vesicles. Five rabbit SR preps and four horse SR preps were electrophoresed on Laemmli SDS-PAGE and stained with Coomassie blue. The amount of protein loaded was 15 µg per lane. The molecular mass of protein gel markers (kDa) is indicated on the left. Gel bands of SERCA and CASQ are identified on the right.

We recently developed a new protocol for isolating SR vesicles from horse muscle with Ca^2+^-dependent ATPase activity by SERCA that was >five-fold greater than previously reported [11]. Here, electrophoretic analysis was performed to corroborate the identification of SR protein distribution in horse muscle SR (Figure 1). Coomassie densitometry was used to determine that SR vesicles from the horse gluteal muscle express ~55% less (53 ± 7%, *p* = 0.005) of the relative content of SERCA compared to rabbit SR (Figure 1, Table S2), although this determination may be a slight overestimate, since horse SR vesicles probably contain a small amount of GP and/or additional proteins that co-migrate with SERCA at ~100 kDa on SDS-PAGE [11]. Our prior quantitative immunoblotting compared the relative amount of SERCA protein in SR vesicles from the horse versus rabbit muscle. For standardization, the SERCA content of SR vesicles from rabbit fast-twitch (white) muscle has been determined to be 55–70% of the total protein (weight/weight), i.e., 5.0–6.4 nmol SERCA per mg of the total SR protein [41,42,43,44]. Immunoblotting with anti-SERCA1 mAb VE12_1_G9 demonstrated that SR vesicles from the horse gluteal muscle express ~35% of the relative amount of SERCA1 as compared to rabbit SR, although this is probably a slight underestimate of the total SERCA content (because horse SR also contains a minor amount of SERCA2, which is incompatible with the use of anti-SERCA1 mAb VE12_1_G9) [9,11].

Despite the minor limitation of each gel-based method, quantitative immunoblotting and Coomassie densitometry are two orthogonal assays that provide similar results, indicating that horse SR vesicles express 35–55% of the SERCA content of rabbit SR vesicles. Based on these two detection methods, we estimate that horse SR contains 45 ± 7% of the SERCA level of rabbit SR. Assuming that (i) the expression level of SERCA in rabbit SR is 6.0 nmol SERCA/mg SR protein, a commonly used mean value, and (ii) horse SR contains 45 ± 7% of the amount of SERCA protein as rabbit SR, then the density of SERCA expressed in horse SR is calculated as 2.7 ± 0.6 nmol SERCA/mg SR protein.

Previously, it has been determined that a rabbit extensor digitorum longus muscle homogenate contains 0.4 ± 0.1 SLN/SERCA (molar ratio) and a rabbit soleus muscle contains 0.9 ± 0.2 [13]. Additionally, SR purified from the pooled rabbit fore and hind leg muscles SR contains 1.2 SLN/SERCA [44] and the contralateral fast-twitch skeletal muscle contains 0.8 SLN/SERCA (molar ratios) [17]. Furthermore, we have used quantitative immunoblotting with purified protein standards to determine that horse SR vesicles express SLN/SERCA at a molar ratio of 0.06 [10]. These previous immunoblot evaluations of the SLN content of the horse SR used a custom antibody raised against a six-residue peptide corresponding to the N-terminus of SLN from a horse. This antibody against the horse sequence detected horse SLN, and a low SLN/SERCA molar ratio in the horse gluteal muscle SR and homogenate [11]. For the present study, we made a new anti-SLN antibody based on the horse N-terminal sequence. Using this antibody, we confirmed that the SR vesicles used in this study contain only trace levels of SLN (Figure S1), as shown before [10,11].

Multiple SR preps from individual horses and rabbits were assayed using densitometry and immunoblotting, demonstrating that there are only slight variations in the high content of key SR proteins (e.g., SERCA and CASQ) and in the low content of contaminating proteins (e.g., myosin) among the multiple SR preparations from each animal (Figure 1). Our next goals are to increase the purity of horse SR vesicles and to increase the number of SR preps purified from horse and rabbit muscles, in order to account for individual variation(s) in muscle proteogenomics. In this report, we detected a small amount of uncertainty in the measured SERCA content (Table S2), yet, as demonstrated below, this uncertainty does not affect the reported results; for example, horse SR vesicles have an enhanced oxalate-facilitated Ca^2+^ transport compared to rabbit SR vesicles, as determined even without normalization to SERCA content.

### 3.2. Horse SR Vesicles Contain an Abundant Level of CASQ Protein, Similar to Rabbit SR Vesicles

CASQ is a high-capacity Ca^2+^-binding protein enriched in the SR lumen SR. The CASQ1 isoform is expressed in fast-twitch skeletal muscles such as horse gluteus and rabbit leg and back muscles, plus mammalian slow-twitch skeletal muscles [9,11,45,46]. The Coomassie staining of the sets of horse preps and rabbit preps analyzed in this study indicates that horse SR contains ~20% more CASQ protein than rabbit SR (Figure 1, Table S2). SDS-PAGE gels labeled with the Stains-all dye corroborated similar levels of CASQ protein expression in horse and rabbit SR [11], assuming that horse and rabbit CASQ orthologs bind the Stains-all dye with similar affinity and metachromatic effect, per molecular electronegativity. Both horse and rabbit CASQ1 have the same predicted isoelectric point of 3.8, as determined using the Isoelectric Point Calculator program [47]. The CASQ-to-SERCA ratio in horse SR vesicles (0.55 ± 0.01) is ~2.25-fold greater (*p* = 0.0001) than in rabbit SR vesicles (0.24 ± 0.03), as determined using in-gel Coomassie densitometry (Figure 1, Table S2). We propose that the relatively high level of CASQ protein in horse SR vesicles contributes to the increased SR Ca^2+^ cycling in vivo and enhanced muscular performance in horses.

### 3.3. Horse SR Vesicles Show Greater ATP-Dependent Ca^2+^ Transport Than Rabbit SR Vesicles

**Figure 2:**
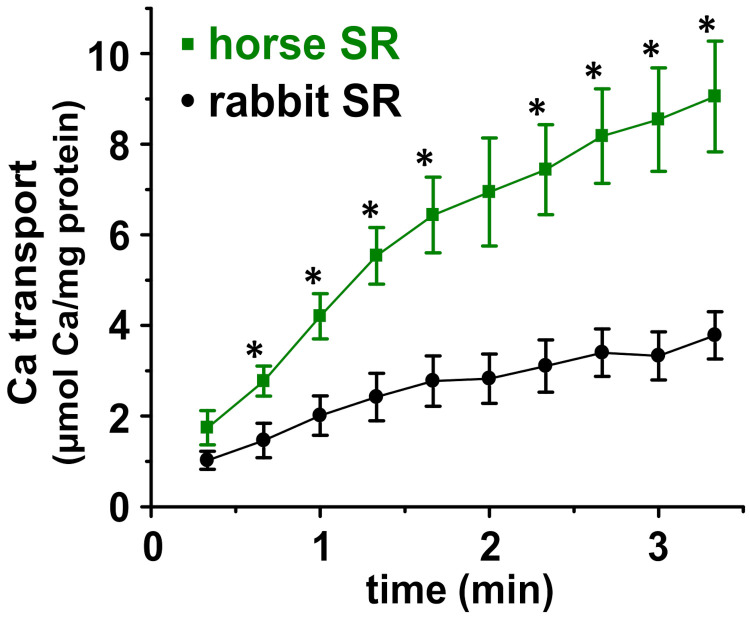
Calcium transport by SR vesicles purified from horse or rabbit muscle. ^45^Ca transport activity was measured at 25 °C in the presence of the Ca^2+^-precipitating anion oxalate, with saturating concentration of substrates (Ca^2+^, Mg^2+^, and ATP), thereby providing a steady-state V_max_ assay. *n* = 4–5 for horse and *n* = 5 for rabbit SR. * significantly different between horse and rabbit using unpaired Student’s *t*-test, *p* < 0.05.

We used our new protocol for isolating horse SR vesicles [11], which show greatly enhanced Ca^2+^-ATPase activity, to assess Ca^2+^ transport by SERCA in horse SR (Figure 1 and Figure 2). ATP-dependent Ca^2+^ transport was measured using a radiometric filtration assay. ^45^Ca^2+^ transport was measured at 25 °C under the V_max_ condition, i.e., in the presence of the following saturating concentrations of substrates: 3 mM ATP, 3.3 mM MgCl_2_, and an ionized Ca^2+^ concentration ([Ca^2+^]_i_) of ~2.4 µM (which was set using an EGTA/Ca^2+^ buffering system). Oxalate and azide were added at 5 mM each. Oxalate is a Ca^2+^-precipitating anion that diffuses into SR vesicles through SR-specific anion/oxalate channels, and thus an oxalate was added to the ^45^Ca^2+^ transport assay to remove the product-inhibition of SERCA using a high concentration of accumulated luminal Ca^2+^ [48,49,50]. Due to the high density of SERCA in muscle SR (~33,000 SERCA molecules per membrane µm^2^) [51], the lumen of SR becomes saturated with Ca^2+^ (>1 mM) within 1–2 turnover cycles of SERCA molecules, as assessed using in vitro assays of SR vesicles and using myocyte-based relaxation assays in the absence of an oxalate [52,53]. The addition of oxalate to in vitro assays allows Ca^2+^ transport by SERCA in SR vesicles to proceed for minutes, instead of second(s), thereby providing a steady-state biochemical measurement of SERCA activity (e.g., in vitro V_max_ assay, as reported in Figure 2 here).

Oxalate-facilitated ^45^Ca^2+^ transport assays have been optimized extensively for SR vesicles, and the current study of SERCA in horse SR vesicles is based on insights reported by these foundational studies [36,48,49,53,54]. Under the assay conditions used in the present study (see Section 2.5), the Ca^2+^ uptake by SR vesicles from horse muscle (i.e., the 10,000× *g* pellet) is specific to SR vesicles, because (i) Ca^2+^ uptake by mitochondria is inhibited by the addition of azide, which collapses the electric potential of the mitochondrial inner membrane [48], and because (ii) the Ca^2+^ uptake by sarcolemmal (SL) vesicles is not facilitated by the addition of oxalate, since SL vesicles lack the SR anion/oxalate channel [49]. The specificity of ATP-mediated Ca^2+^ transport by SERCA was further demonstrated by control experiments in (i) the absence of ATP, which eliminated >95% of the Ca^2+^ transport by horse SR vesicles, and (ii) the presence of thapsigargin (TG), a SERCA-specific inhibitor [55] that eliminated >95% of the Ca^2+^ uptake by horse SR vesicles.

The in vitro ^45^Ca^2+^ filtration assay demonstrated that the initial rate of Ca^2+^ transport by horse and rabbit SR vesicles is linear for ~60–80 s at 25 °C (Figure 2). Using linear regression of four time points (0, 20, 40, and 60 s), horse SR vesicles exhibit a Ca^2+^ transport rate of 4.2 ± 0.7 µmol Ca^2+^/mg protein/min (i.e., international unit, IU), while rabbit SR vesicles exhibit a Ca^2+^ transport rate of 1.8 ± 0.5 IU (Figure 2). These assay conditions, via an oxalate-facilitated uptake, demonstrated that horse SR vesicles show a 2.3 ± 0.7-fold greater rate of Ca^2+^ transport than rabbit SR vesicles, even though horse SR vesicles contain 45 ± 7% of the SERCA protein content of rabbit SR vesicles, as determined using Coomassie densitometry (Figure 1) and quantitative immunoblotting [11]. Similarly, horse SR vesicles show 52% of the Ca^2+^-activated ATPase activity compared to rabbit SR vesicles (Table 1) [11].

The amount of oxalate-facilitated Ca^2+^ accumulation by horse and rabbit SR vesicles continued to increase until ~5 min, when the rate of net ^45^Ca^2+^ uptake reached zero, i.e., a plateau of the steady-state accumulation of ^45^Ca^2+^, where the uptake and release/leak rates are equal. At ~5–10 min, the total amount of accumulated intravesicular Ca^2+^ began to decrease, probably due to the disruption of SR vesicle integrity by the growth of luminal Ca^2^/oxalate deposits sized beyond a vesicular capacity.

The robust rate of Ca^2+^ transport by horse SR vesicles indicated that horse SR vesicles in the 10KP fraction, isolated by our newly developed protocol [10,11], are mostly intact and sealed. Table 1 reports that the Ca^2+^ transport V_max_ rate of SR vesicles isolated from horse muscle [5,56,57,58,59]. Here, we show that the Ca^2+^ transport V_max_ rate by horse SR vesicles, as isolated using the improved protocol [10,11], is 8–23-fold greater than the Ca^2+^ transport V_max_ rate of horse SR vesicles isolated by previously reported protocols (Table 1). Thus, we have demonstrated that SR vesicles from horse muscle provide a useful system for studying Ca^2+^ transport by horse SERCA. We propose that the high V_max_ rate of Ca^2+^ transport by horse SERCA is facilitated by the lack of SLN regulatory peptides [10,11] and by the abundance of CASQ protein in horse SR (Figure 1) [9,11].

### 3.4. Horse and Rabbit SERCA Show Similar Temperature-Dependence of Ca^2+^-Activated ATPase Activity

**Figure 3:**
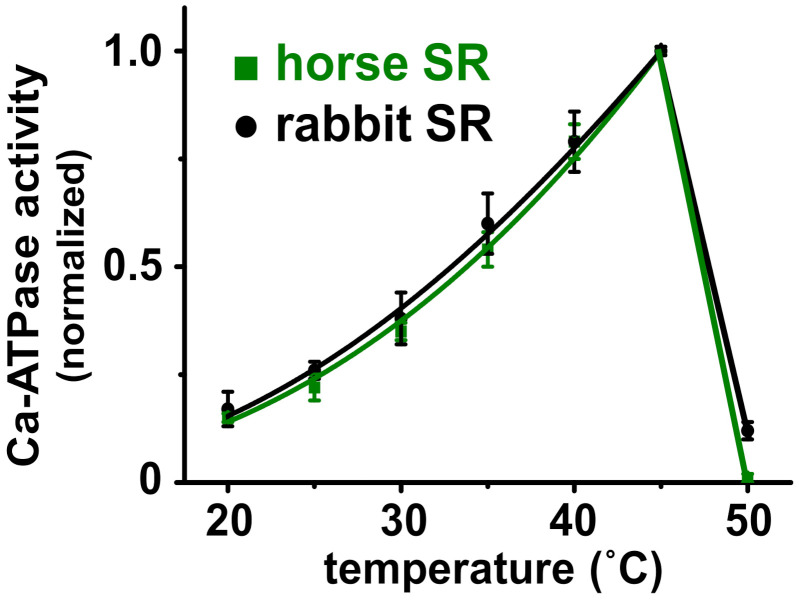
Horse and rabbit SERCA show a similar temperature dependence of Ca^2+^-activated ATPase activity. SR vesicles from horse and rabbit muscle were assayed for ATP hydrolysis in the presence of a saturating concentration of substrates (100 µM Ca^2+^ and 5 mM Mg-ATP), in the presence of Ca^2+^ ionophore A23187.

The internal temperature of actively contracting horse muscle is 42–44 °C [60]. Since temperature controls the structure and activity of enzymes, we compared the temperature dependence of the Ca^2+^-activated ATPase V_max_ activity for SERCA in horse and rabbit SR (Figure 3). With a saturating concentration of substrates, both horse and rabbit SERCA show a biphasic effect of enzyme activation and inactivation, with a robust ~six-fold increase in activity when raising the temperature from 20 to 45 °C using 5 °C steps. This is followed by a steep ~19-fold decrease in activity when raising the temperature from 45 to 50 °C (Figure 3). The 50% transition temperature from peak activity to thermal inactivation (T_i_) is ~48 °C for both horse and rabbit SERCA. For comparison, rat SERCA in SR vesicles from rat skeletal muscle exhibits a 50% thermally induced inactivation at T_i_ = 47 ± 0.7 °C [61]. The similarities in the thermal activation and the inactivation of SERCA from horse and rabbit muscle indicate a similarity in the protein structure and function of the two orthologs, such that global structure and temperature-dependence probably do not account for the increased relative rate of Ca^2+^ transport by horse SERCA. The temperature-dependence assay also demonstrates that horse SERCA in the presence of a low level of horse SLN shows a similar biphasic thermal profile of Ca^2+^-ATPase activity to rabbit SERCA in the presence of an approximately equimolar level of rabbit SLN [17,44]. We propose that the robust Ca^2+^ transport activity of SERCA in horse SR vesicles compared to rabbit SERCA is due, in part, to the lack of SLN inhibition, and possibly due to specific variations in the amino acid sequence and local secondary structural elements of horse SERCA.

### 3.5. Horse and Rabbit SERCA Show Similar Ca^2+^-Dependent Cleavage by Proteinase K

**Figure 4:**
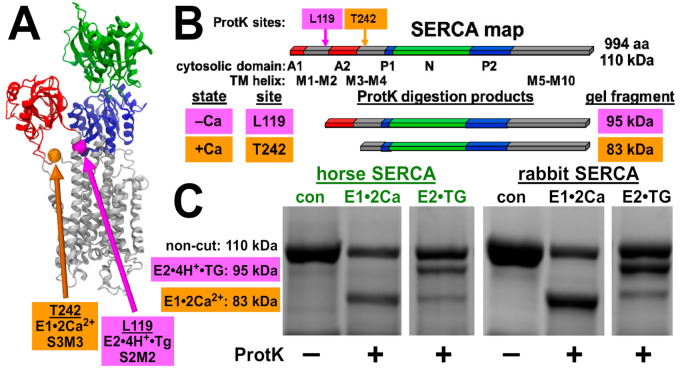
Horse and rabbit SERCA show similar calcium-dependent cleavage by Proteinase K. (**A**), location of conformation-specific ProtK sites shown in the X-ray crystal structure of rabbit SERCA in the calcium-free E2•TG state (PDB ID code 1IWO [62]). (**B**), location of conformation-specific ProtK sites in the primary topology map of rabbit SERCA. (**C**), horse and rabbit SR vesicles were digested with ProtK, and proteolytic fragments of SERCA were analyzed using SDS-PAGE and Coomassie staining. The same cleavage pattern is observed between the horse and the rabbit. The molecular mass of SERCA and diagnostic ProtK fragments are indicated on the left. SERCA samples were electrophoresed on the same Coomassie gel, and the gel slices shown are presented with the same image scale of absorbance intensity for Coomassie-stained bands.

To assess the structural dynamics of horse SERCA, we utilized controlled Proteinase K (ProtK) cleavage to assess the conformation-specific cleavage of SERCA. ProtK shows selectivity for accessible sites in protein segments that exhibit enhanced structural dynamics prior to ProtK binding and cleavage. ProtK cleaves SERCA at Actuator domain linker segments in the membrane/water interfacial boundary [39,40], and these sites have been well characterized for the rabbit SERCA1 protein. In the presence of Ca^2+^ (E1•2Ca^2+^ biochemical state), ProtK cuts rabbit SERCA selectively at residue T242 on stalk segment three leading out of the Actuator domain into transmembrane segment three, yielding a major C-terminal fragment comprising residues 243–994 (molecular mass of 83 kDa) (Figure 4). In the absence of Ca^2+^ (E2•TG biochemical state, stabilized by the inhibitor thapsigargin), ProtK cuts rabbit SERCA selectively at residue L119 on stalk segment two, yielding a major C-terminal fragment comprising residues 120–994 (molecular mass of 95 kDa), with a low amount of secondary cleavage at T242, i.e., also producing a minor fragment of 83 kDa (Figure 4). As in the amino acid sequence of rabbit SERCA, horse SERCA also encodes residues L119 and T242.

Here, ProtK digestion demonstrated that horse SERCA shows a similar Ca^2+^-dependent conformational change as rabbit SERCA, providing similar fragment intensities of the 83 and 95 kDa bands as rabbit SERCA in the presence and absence of Ca^2+^, respectively. Thus, the ProtK assay demonstrates that horse SERCA in the absence of SLN shows a similar Ca^2+^-dependent conformation change as rabbit SERCA in the presence of SLN. We propose that (i) the structural dynamics of stalk segments two and three of horse SERCA is similar to that of stalk segments two and three of rabbit SERCA, and (ii) the absence of SLN as a SERCA-bound protein subunit does not affect the equilibrium of Ca^2+^ bound versus Ca^2+^ free states of horse SERCA in ligand-induced intermediates, i.e., in a non-cycling enzyme stabilized in E1•2Ca^2+^ or E2•TG. We conclude that the ProtK assay demonstrates similarity in the structural dynamics of horse and rabbit SERCA, suggesting that the lack of SLN in horse SR helps enhance Ca^2+^ transport by horse SERCA.

As further validation of the new protocol for purification of horse SR, the ProtK assay provides an almost complete digestion of horse and rabbit SERCA (110 kDa bands) in 15 min at 23 °C; thus, almost all of the SR vesicles in horse and rabbit preps are oriented right-side out, with an extravesicular location of the SERCA headpiece, as expected. We conclude that the reported protocol for isolation of SR vesicles from horse muscle provides a preparation suitable for functional and structural comparisons of horse SERCA to orthologs such as rabbit and human enzymes [36,63,64].

## 4. Discussion

The present study provides a comparative assessment of Ca^2+^ transport proteins from horse and rabbit muscle, thereby providing quantitative measurements of fundamental parameters of SR function at the molecular level. Our specific purpose was to analyze SR Ca^2+^ transport in horses, a species that has long been selected for speed by evolution and breeding. Here, we report the biochemical analysis of Ca^2+^ transport in horse SR vesicles, with comparison to widely used experimental models of SERCA and SR from rabbit muscle. The results provide new insights into Ca^2+^ transport regulations in horse muscle, including proposed mechanisms for high muscular performance.

### 4.1. Analysis of SERCA Ca^2+^ Transport and ATPase Activities in SR Vesicles from Horse and Rabbit Muscle

We compared SERCA activity from horse gluteus and rabbit skeletal muscles using a radiometric assay of Ca^2+^ transport. SERCA product-inhibition by accumulated luminal Ca^2+^ was relieved by an addition of the Ca^2+^-precipitating anion oxalate in the transport assay. We previously showed that the maximal rate of total Ca^2+^-activated ATPase activity of SERCA was lower in horse SR vesicles than in rabbit SR vesicles (measured per mg of SR protein), and that the specific activity (corrected for the relative content of SERCA per mg of SR protein) of horse SERCA was equal to or greater than that of rabbit SERCA [11]. Here, we report that the maximal rate of total ATP-dependent Ca^2+^ transport for horse SR vesicles is 2.3-fold greater than the Ca^2+^ transport V_max_ measured for rabbit SR vesicles (mg horse SR protein/mg rabbit SR protein) (Figure 2), even though the SERCA content in SR vesicles is ~two-fold lower for the horse than the rabbit (Figure 1) [11].

The differences in the V_max_ of the horse versus rabbit SERCA orthologs could potentially be attributed to the lack of SLN peptide in horse muscle [10,11]. Another possibility for increased transport is sequence variation in the horse versus rabbit SERCA proteins that affect the equilibrium distribution among biochemical intermediates, i.e., the activation energy for conformational transitions in steps along the enzymatic cycle [65]. For example, the single-residue variations R164H and R559C—each of which individually decrease the activity of bovine SERCA—are sufficient to induce congenital pseudomyotonia syndrome in cattle [66,67,68].

The horse and rabbit SERCA showed similar activation of ATP hydrolysis in vitro upon increasing the assay temperature from 20 to 45 °C using 5 °C steps (Figure 3), indicating that the two orthologous Ca^2+^ pumps have a similar standard free energy of enzyme activation (E_a_), a key parameter that determines the rate of enzyme activity. Additionally, increasing the assay temperature from 45 to 50 °C inactivated both the horse and rabbit SERCA almost completely, suggesting that the SERCA Ca^2+^ pump from the two species share a similar thermostability, indicating similar energetics of unfolding for key structural element(s) involved in enzyme catalysis. The lack of difference in the temperature dependence of Ca^2+^-ATPase activity by horse and rabbit SERCA (Figure 3) does not support the activity difference due to phospholipids: either directly through altered lipid composition and protein interaction, or indirectly through membrane fluidity and bilayer phase modulation. An additional biochemical assay of the SERCA structure and function used the conformation-specific ProtK cleavage assay (Figure 4), which provided information to help interpret the results from the standard Ca^2+^ transport (Figure 2) and the Ca^2+^-ATPase [11] assays. The ProtK assay revealed a similar pattern of protein cleavage for the horse and rabbit SERCA in Ca^2+^-bound and Ca^2+^-free biochemical states. Thus, this result indicates a similar residue accessibility and structural dynamics on the stalks segments of the actuator domain for the two SERCA orthologs in each specific ligand-stabilized biochemical intermediate (e.g., residue L119 in E2•TG or residue T242 in E1•2Ca^2+^). Although neither of these two biochemical assays provided information on molecular mechanism(s) that produce robust Ca^2+^ transport by horse SR, both assays demonstrated the functional and structural similarities of SERCA orthologs from horse gluteus and rabbit muscles.

### 4.2. The Relative Ratio of Ca^2+^ Transport to ATP Hydrolysis Is Greater for Horse SR Vesicles Than Rabbit SR Vesicles

SLN decreases the energetic efficiency of SERCA activity by partially uncoupling Ca^2+^ transport from ATP hydrolysis, i.e., by decreasing the Ca^2+^/ATP coupling ratio below the maximum ratio of 2 Ca^2+^ ions transported per ATP molecule hydrolyzed [18,20,21,22,23,26]. Table 1 reports the Ca^2+^ transport and ATP hydrolysis activities of horse and rabbit SR vesicles. The following two separate and distinct experimental procedures were utilized to determine each of these activities: (i) an oxalate-facilitated ^45^Ca^2+^ transport assay and (ii) an ionophore-facilitated ATPase assay. Thus, due to the different experiment conditions designed to relieve product inhibition for steady-state biochemical assays (e.g., precipitation of ^45^Ca^2+^-oxalate deposits within SR vesicles vs. an ionophore-mediated release of Ca^2+^ from SR vesicles), calculating the absolute coupling ratio of SERCA was not feasible. However, the relative ratio of the Ca^2+^ uptake per ATP hydrolysis could be calculated as an activity/activity ratio (IU/IU) (see Table 1, Column 4). With this relative ratio of SERCA functions (i.e., a pseudo-coupling ratio), the horse SR vesicles showed a ~1/1 proportion of Ca^2+^ transport to ATPase activities, as determined by four reports measuring both activities (Table 1) [11,56,58,59]. This relative ratio of ~1/1 transport/ATPase in horse SR vesicles was observed using distinct protocols for the isolation of SR vesicles from horse skeletal muscle, even though the horse SR preps showed a range of 5–25-fold difference in the measured activities (Table 1). In comparison, the SR vesicles from rabbit skeletal muscle (which expresses near equimolar levels of SERCA and SLN) showed a relative ratio of ~0.3 transport/ATPase, using the two separate assays for each activity, i.e., determining the pseudo-coupling ratio (Table 1). We propose that SERCA in horse SR vesicles shows a greater apparent Ca^2+^/ATP coupling ratio due to minimal SLN content in the SR membrane [10,11] and an abundant CASQ in the SR lumen (Figure 1) [11].

### 4.3. Proposed Physiological Effects of Minimal Expression of SLN and Abundant Expression of CASQ on Horse Muscular Performance

We hypothesize that the low SLN/SERCA protein ratio in horse SR vesicles enhances the rate of SR Ca^2+^ uptake and the loading level of SR Ca^2+^ stores. Horse SR shows ~two-fold greater rate of Ca^2+^ transport than rabbit SR (Figure 2), even though horse SR expresses a ~two-fold lower SERCA content than rabbit SR (Figure 1) [11]. These results from horse SR are consistent with those from transgenic mouse models in which (i) the knock-out of SLN protein expression in slow-twitch soleus muscle increases the rate of Ca^2+^ transport by SERCA, and (ii) the over-expression of SLN protein in cardiac and skeletal muscle decreases the rate of Ca^2+^ transport by SERCA [27,28,69]. For horse myocytes, the increased rate of Ca^2+^ transport by SERCA and the increased expression level of CASQ suggests that horse SR accumulates, stores, and releases an increased level of total Ca^2+^, thereby producing an ionotropic-like enhancement of muscle contractility [70].

**Figure 5:**
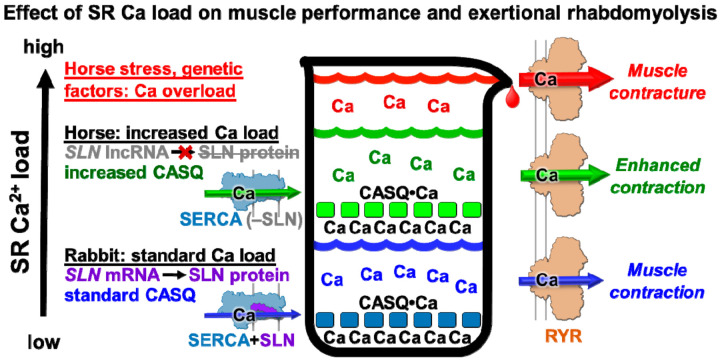
Proposed model for the roles of SR calcium regulation in horse muscle performance and exertional rhabdomyolysis. This schematic diagram illustrates the hypothesis that luminal Ca^2+^ stores and Ca^2+^ cycling in horse myofibers are enhanced by a relatively low ratio of SLN/SERCA and a relatively high ratio of CASQ/SERCA. We propose that high luminal Ca^2+^ promotes a store overload-induced Ca^2+^ release (SOICR) through the RYR Ca^2+^ channel [30,32], which, combined with stress-induced RYR Ca^2+^ leak [71,72], increase the incidence of contracture events in horse muscle [6,7,73,74]. It is possible that the subsequent Ca^2+^-induced activation of proteases, lipases, oxidative stress, and cellular remodeling contributes to the etiology of equine exertional rhabdomyolysis [8].

CASQ is the primary Ca^2+^ storage protein in SR, and CASQ is tightly linked to the regulation of Ca^2+^ cycling in muscle. Since SR Ca^2+^ release is sensitized by luminal Ca^2+^, an increase in luminal Ca^2+^ stores and an associated RYR1 Ca^2+^ release could result in a more rapid and more powerful muscle contraction, i.e., producing a positive inotropic effect in vivo. The correlation of a higher rate of SERCA transport in SR vesicles with an increased rate of clearance of cytosolic Ca^2+^ transient suggests that horse myocytes exhibit a positive lusitropic effect (Figure 5).

Horse breeds have been bred selectively to further enhance their speed and with that has come an incidence of exertional rhabdomyolysis of 3–7% [4,75]. Elevated cytosolic Ca^2+^ has been detected in (a) primary myocytes isolated from horse muscle during an episode of acute exertional rhabdomyolysis [76] and (b) cultured myocytes differentiated from muscle myoblasts obtained from horses susceptible to recurrent exertional rhabdomyolysis [74]. The lack of a protein expression of SERCA regulatory peptides in horse muscle may be a molecular mechanism to produce strong acceleration and rapid muscular relaxation, thereby giving horses an advantage in escaping predators. While slightly elevated cytosolic Ca^2+^ may provide an advantage that enhances muscle power output, excessively elevated cytosolic Ca^2+^ may become a disadvantage that decreases contractility, such as through an SR store overload-induced Ca^2+^ release (SOICR) [32]. We suggest that a heritable crossover between these two contractile conditions may occur following genetic pressure such as performance-based breeding (Figure 5).

We propose that the low SLN/SERCA protein ratio in horse SR vesicles enhances the rate of SR Ca^2+^ reuptake and the loading level of SR Ca^2+^ stores, thereby enabling a more rapid and more powerful contraction of horse gluteal muscle in vivo. An increase in the intrinsic rate of Ca^2+^ transport by horse SERCA (i.e., a possible lack of SLN uncoupling and/or an enhanced V_max_), coupled with a robust level of CASQ expression, may also serve as molecular mechanisms that potentiate SR contributions to functional contractility in horse muscle (Figure 5).

### 4.4. Proposed Role of SLN Expression in Horse Exertional Rhabdomyolysis and Relationship to Human Muscular Dystrophies

With the natural selection of horses as fast-moving prey animals, why would they evolve to have a unique SLN amino acid sequence and lack of expression that are not present in closely related ungulates such as rhinoceros? One possible advantage for the lack of a SERCA regulatory peptide inhibitor includes a genetic improvement that generates an increased luminal Ca^2+^ load to provide a greater ability to escape predators due to stronger acceleration and stronger contraction. After breeding over millennia, could enhanced Ca^2+^ cycling and contractile abilities have been selected to the extreme in some breeds, with *SLN* RNA upregulated and SLN protein downregulated? Thoroughbred horses with recurrent exertional rhabdomyolysis (RER) have 50% faster relaxation times than horses that are not predisposed to recurrent exertional rhabdomyolysis [73]. Furthermore, horses with recurrent exertional rhabdomyolysis have faster racing speeds than horses that are not predisposed to exertional rhabdomyolysis [3]. Thus, two parameters of horse muscular performance are associated with horse susceptibility to RER.

In human muscular dystrophies, the effect of modulating the SLN expression level and the inhibitory function is unknown. In compelling mouse and canine models, enhancing SLN protein expression has been proposed as an effective therapy, as reported by Babu et al. [77]. Indeed, SLN gene expression and protein levels are increased many-fold in standard mouse models of Duchenne muscular dystrophy, e.g., *mdx* and *mdx*/*utr*-*dko* [28]. However, decreasing the SLN expression in mouse models is reported to have beneficial or deleterious effects, depending on the genetic model and etiology studied [77,78,79]. To help delineate these disparate effects, additional correlations should be determined between Ca^2+^ transport regulation and muscular performance in animal models and human disease, which could then provide insights on the utilization of species-dependent mechanisms for contractile and therapeutic control.

## 5. Conclusions

Our integrative cellular, transcriptional, and biochemical methodologies [9,10,11,80,81] provide novel information on SERCA activity in horse SR, which is a unique physiological system with high-capacity Ca^2+^ transport. The results reported here contribute to the understanding of Ca^2+^ cycling in myocytes, with relevance to muscular performance, adaptability, and disease. The results shown here provide a foundation to further dissect the mechanistic roles of SERCA, CASQ, and SLN in horse muscle contractility, including correlations with gene and protein expression levels. We propose that analyses of horse Ca^2+^ transport will enhance the basic understanding and therapeutic utilization of Ca^2+^ control in muscle contractility.

## Figures and Tables

**Table 1:** Reported Ca^2+^-ATPase and Ca^2+^ transport activities in SR vesicles purified from horse muscle. Published results of horse SERCA activities are listed from studies using unfractionated SR vesicles purified from horse muscle, as assessed using the oxalate-facilitated ^45^Ca^2+^ transport assay (Figure 2) or the ionophore-facilitated Ca^2+^-activated ATPase assay [11]. Our improved protocol for isolating horse SR vesicles from gluteal muscle provided a Ca^2+^ transport activity of 4.2 ± 0.7 IU at 25 °C and a Ca^2+^-activated ATPase activity of 4.0 ± 0.4 IU at 37 °C. Prior to this study, the maximum reported activities of horse SR vesicles were an oxalate-facilitated Ca^2+^ transport activity of 0.55 ± 0.16 IU at 37 °C and an ionophore-facilitated Ca^2+^-activated ATPase of 0.73 ± 0.14 IU at 37 °C. For comparison, Table 1 also lists the Ca^2+^ transport and Ca^2+^-ATPase activities of unfractionated SR vesicles purified from rabbit fast-twitch muscle, assayed under the same conditions used for horse SR vesicles in this study (Figure 2) and [11], respectively.

SR	Ca^2+^ Transport (IU) + Oxalate ^a^	Ca^2+^-ATPase (IU) + A23187 ^b^	Transport/ATPase (IU/IU)
Horse (*Anal. Biochem.* 2020 ^c^, this study ^d^)	4.2 ± 0.7 ^d^	4.0 ± 0.4 ^c^	1.05 ± 0.20
Horse (*Eq. Vet. J. Suppl.* 1998 ^e^)	0.18 ± 0.02	0.16 ± 0.01	1.13 ± 0.13
Horse (*J. Anim. Sci.* 1995 ^f^)	0.19 ± 0.02	0.16 ± 0.01	1.19 ± 0.12
Horse (*J. Appl. Physiol.* 1989 ^g^)	0.55 ± 0.16	0.73 ± 0.14	0.75 ± 0.35
Rabbit ^h^	1.8 ± 0.5 ^d^	7.6 ± 0.5 ^c^	0.24 ± 0.14

^a^ Oxalate, a Ca^2+^-precipitating anion, was added to the ^45^Ca^2+^ transport assay in order to trap the accumulated intravesicular Ca^2+^. ^b^ A23187, a Ca^2+^ ionophore, was added to the Ca^2+^-activated ATPase assay in order to release the accumulated intravesicular Ca^2+^. ^c^ Data are from Autry et al. [11]. ^d^ This study (Figure 2), respectively. ^e^ Wilson et al., *Eq. Vet*. *J*. *Suppl*. 1998 [59]. ^f^ Wilson et al., *J*. *Anim*. *Sci*. 1995 [58]. ^g^ Byrd et al., *J*. *Appl*. *Physiol*. 1989 [56]. ^h^ SR vesicles from rabbit fast-twitch skeletal muscle purified by slight adaptation [11] of the protocol from Ikemoto et al., *J. Biol. Chem.* 1971 [36].

## Data Availability

Data supporting the findings of this study are available within the article and/or its Supplementary Material. Full raw data sets are available from the corresponding authors, upon reasonable request.

## Supplementary Materials

The following are available online at https://www.mdpi.com/article/10.3390/vetsci8120289/s1, Figure S1: SLN Western blot of SR vesicles using a novel, custom-ordered anti-horse-SLN polyclonal antibodies pAb 3378, Table S1: Horse muscle tissue samples, Table S2: Densitometry data for Coomassie gel.
