# Supplementary material for: Sarcoplasmic Reticulum from Horse Gluteal Muscle Is Poised for Enhanced Calcium Transport
Source: Vet Sci. 2021 Nov 23;8(12):289. doi: 10.3390/vetsci8120289 (PMC8705379; doi:10.3390/vetsci8120289)
Supplement: Supplementary file 1 [file vetsci-08-00289-s001.zip › vetsci-1449185-supplementary.pdf]

SUPPLEMENTAL DATA

Sarcoplasmic reticulum from horse gluteal muscle is poised for enhanced calcium transport

Joseph M. Autry [1], Bengt Svensson [1], Samuel F. Carlson [1], Zhenhui Chen [2],
Razvan L. Cornea [1], David D. Thomas [1,†], Stephanie J. Valberg [3,†]

[1] University of Minnesota, Dept. of Biochemistry, Molecular Biology, and Biophysics
[2] Indiana University School of Medicine, Dept. of Medicine, Krannert Institute of Cardiology
[3] Michigan State University, Dept. of Large Animal Clinical Sciences, McPhail Equine Performance Center
[†] Co-Senior Authors

p. S1 **Table of Contents**

p. S2 **Table S1.** Horse muscle tissue samples.

p. S3 **Table S2.** Densitometry data for Coomassie gel.

p. S4 **Figure S1**. SLN Western blot of SR vesicles using a novel, custom-ordered anti-horse-SLN polyclonal antibodies pAb 3378.

p. S5 **References**

Table S1: List of horse muscle tissue samples.

Animal Number	Source	Species Breed	Sex	Age	Husbandry
9560	Owner donation	Quarter horse	Male, castrate	10	Stall/pasture
9623	Owner donation	Quarter horse	Female	18	Stall/pasture
9657	Owner donation	Thoroughbred	Male, castrate	10	Stall/pasture
12121	Owner donation	Quarter Horse	Male, castrate	16	Stall/pasture

Table S2: Densitometry data for Coomassie gel.

Sample	SERCA band density	CASQ Band Density	Ratio CASQ/SERCA:
Rabbit 1	1873216	541632	0.29
Rabbit 2	1859840	648192	0.35
Rabbit 3	2588032	596736	0.23
Rabbit 4	2964864	582464	0.20
Rabbit 5	2695296	537984	0.20
Horse 1	1168000	628480	0.54
Horse 2	1389824	713856	0.51
Horse 3	952192	549440	0.58
Horse 4	1561152	880960	0.56

The Coomassie-stained gels were analyzed to quantitate the relative absorbance of the SERCA and CASQ protein band using a GelDoc EZ imaging system with the software Image Lab 5.0 (Bio-Rad Laboratories Incorporated; Hercules, CA). The amount of SERCA in horse SR is 0.53 ± 0.07 (p=0.005) times the amount of SERCA in rabbit SR, i.e. ~45% lower. The CASQ amount in horse SR is 1.19 ± 0.11 (p=0.21) times the rabbit amount, i.e ~20 % higher. The average ratio for CASQ to SERCA was in rabbit 0.24 ± 0.03 and in horse 0.55 ± 0.01 (p=0.0001), i.e. a 2.25 ± 0.25 times higher CASQ/SERCA ratio.

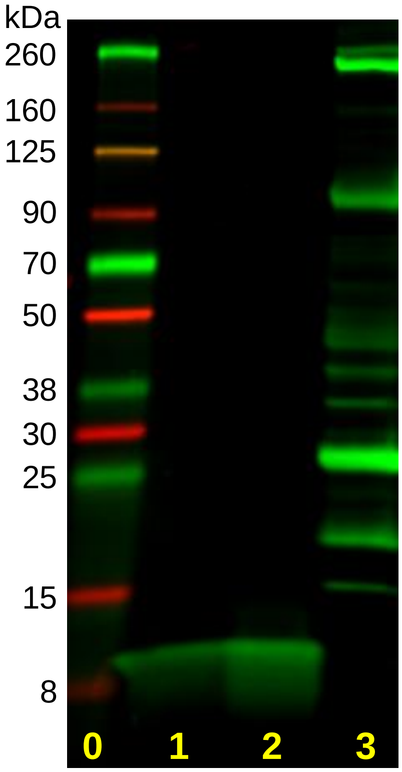

Figure S1. Immunoblot of SR vesicles using the new anti-horse-SLN polyclonal antibody pAb 3378. We raised a polyclonal antibody against horse SLN (pAb GS3378) to validate previously published immunoblot results (1,2). This primary antibody was custom ordered from Genscript (Piscataway, NJ) using a peptide containing horse SLN residues ^{1}MEWRRE^{6} as the immunogen. Samples were electrophoresed through a CriterionTM Precast Gel (4-20% TGX, 18 Well Comb, 30 µL; Cat #: 567-1094) and immunoblotting was performed as previously published (2). Lane 0: 3 µL Chameleon SDS-PAGE standard (Li-Cor # 928-60000). Lane 1: 0.01 µg Horse SLN standard. Lane 2: 0.025 µg Horse SLN standard. Lane 3: 10 µg horse SR (10K). The results were the same as with previous antibodies i.e., only very low levels of horse SLN were detected in horse SR vesicles.

REFERENCES

1. Autry, J. M., Karim, C. B., Cocco, M., Carlson, S. F., Thomas, D. D., and Valberg, S. J. (2020) Purification of sarcoplasmic reticulum vesicles from horse gluteal muscle. *Anal Biochem* **610**, 113965
2. Autry, J. M., Karim, C. B., Perumbakkam, S., Finno, C. J., McKenzie, E. C., Thomas, D. D., and Valberg, S. J. (2020) Sarcolipin Exhibits Abundant RNA Transcription and Minimal Protein Expression in Horse Gluteal Muscle. *Vet Sci* **7**, 178
